# Supplementary material for: Investigation of Chemical Compositions and Biological Activities of Mentha suaveolens L. from Saudi Arabia
Source: Molecules. 2022 May 5;27(9):2949. doi: 10.3390/molecules27092949 (PMC9099798; doi:10.3390/molecules27092949)
Supplement: Supplementary file 1 [file molecules-27-02949-s001.zip › molecules-1685651-supplementary.pdf]

Investigation of Chemical Compositions and Biological Activities of *Mentha suaveolens* L. from Saudi Arabia

Supplementary Data and Materials

2.3. Analysis of volatile oils of Mentha *by GC and GC/MS*

2.3.1. GC analysis

About two microliters of each pure volatile oils were used. The oils were analyzed by using Hewlett-Packard model 5890 (Hewlett-Packard, Perkin elmer Co., USA) equipped with a flame ionization detector and a fused silica capillary column DB-5 (Zebron Co., USA) (60 m × 0.32 mm, internal diameter). Initially, the oven temperature was maintained at 50°C for 5 min, then raised from 40 to 260°C at 3°C/min. At a flow rate of 1.1 mL/min, helium was used as the carrier gas. Injector and detector temperatures were set at 220 and 250°C, respectively. The retention indices (Kovats index) of the separated volatile constituents were determined using hydrocarbons (C7:C21; Sigma–Aldrich Co.) [13].

2.3.2. GC/MS analysis

A coupled GC Hewlett-Packard model 5890/MS Hewlett-Packard MS 5970 (Hewlett-Packard) was used to perform the analysis. The ionization voltage was 70 eV, and the mass range obtained m/z was 39–400 a.m.u. The isolated peaks were determined using data from the mass spectra library (National Institute of Standard and Technology) and correlated with those of authentic compounds and published data. The quantitative determination was performed based on peak area integration. The chemical components of volatile oils were identified by comparing their mass spectra with those in NIST Library, retention indices (RI), standard compounds, and literature data [13,14,16,18,20].

2.4. HPLC Analysis of non-Volatile Compounds

An Agilent 1100 model HPLC system equipped with an Phenomenex Aqua C18 (5 µm) 250mm × 4.6 mm i.d. column (Torrance, CA)) and a diode array detection system G1315A was used to analyze the ethanolic extracts. The mobile phase consisted of 3.5% acetic acid in water (A) and 3.5% acetic acid in methanol (B). The gradient mode was started at 20% B, then increased linearly to 100% B within 40 min, and held to the end (40 to 85 min). For all samples, the injection volume was 100 µL. The UV detector was set at 280-360 nm and the flow rate of the mobile phase was 1 mL/min [58].

All phenolic standards were prepared in methanol within a range from 0.5 to 100 µg/mL and these standards were used for quantitative and qualitative identification of phenolic compounds in M. suaveolens L extract chemical composition depending on the standards calibration curve and retention time, respectively.

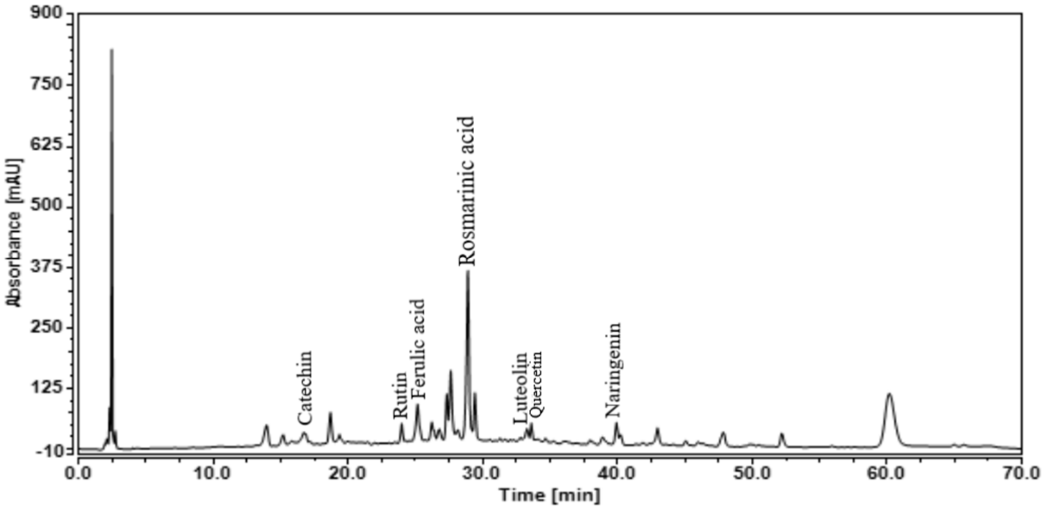

Figure S1. Chemical composition in whole plant of fresh Mentha analyzed by using HPLC.

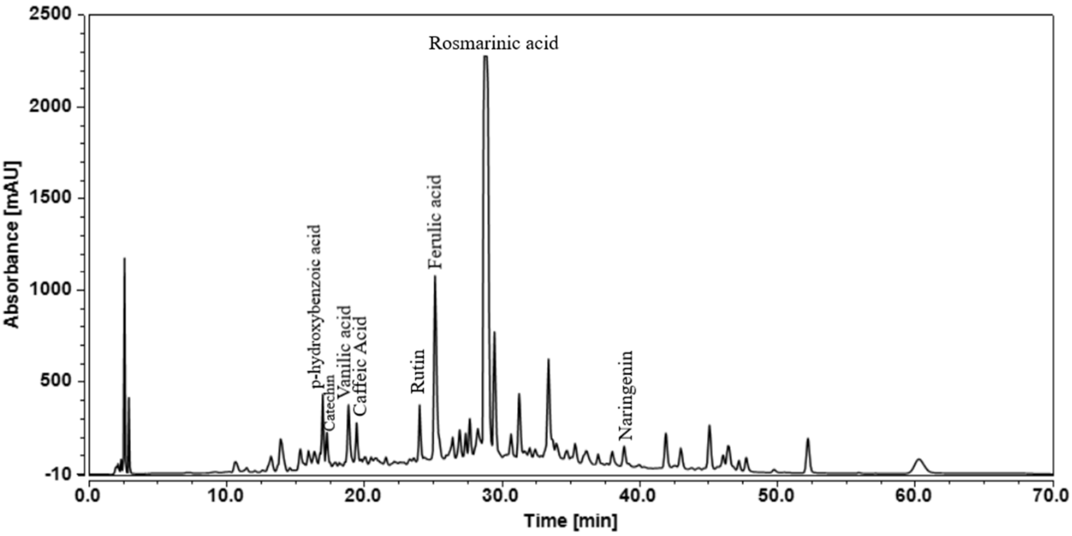

Figure S2. Chemical composition in leaves of fresh Mentha analyzed by using HPLC.

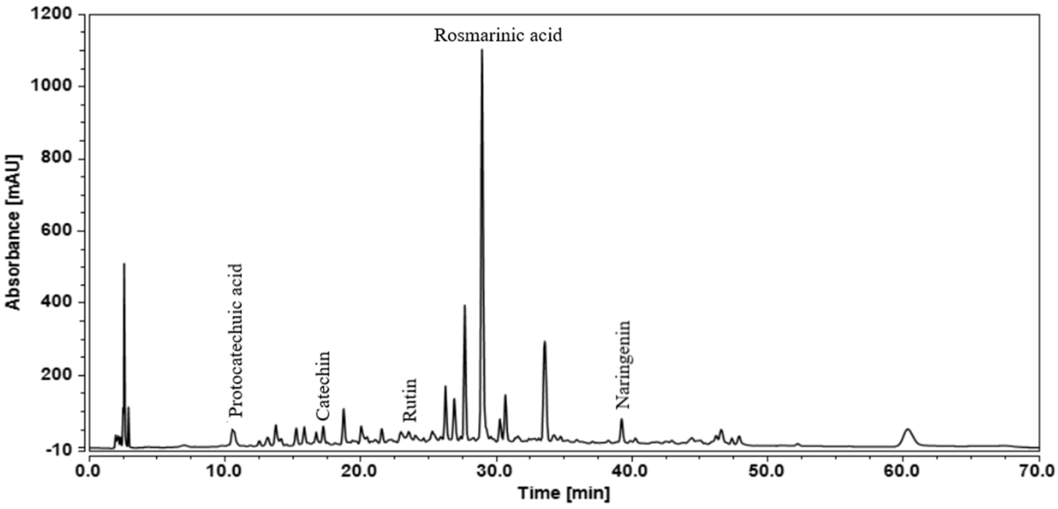

Figure S3. Chemical composition in stems of fresh Mentha analyzed by using HPLC.

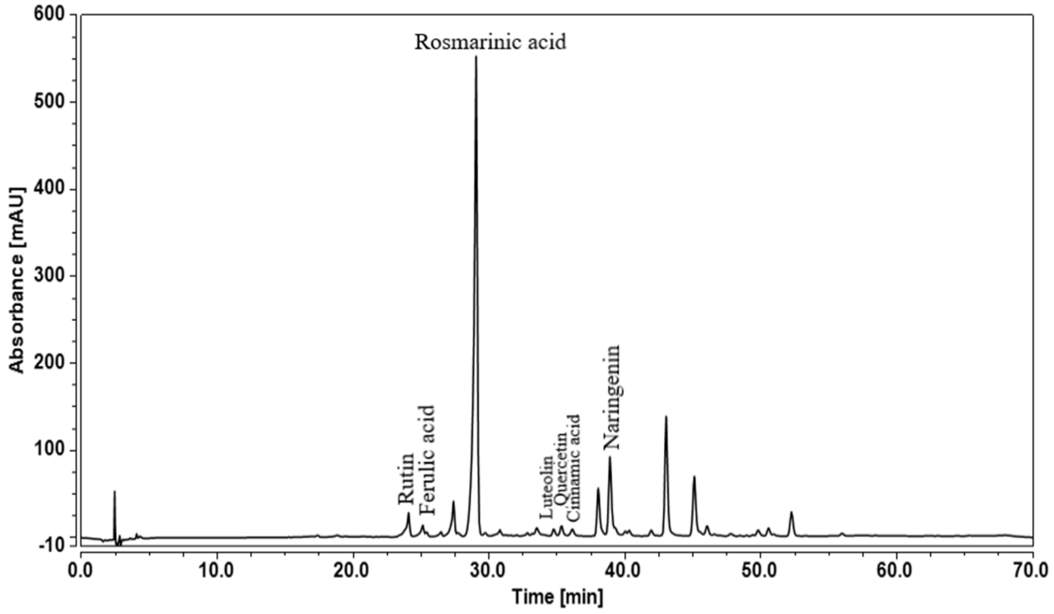

Figure S4. Chemical composition in whole plant of dried Mentha analyzed by using HPLC.

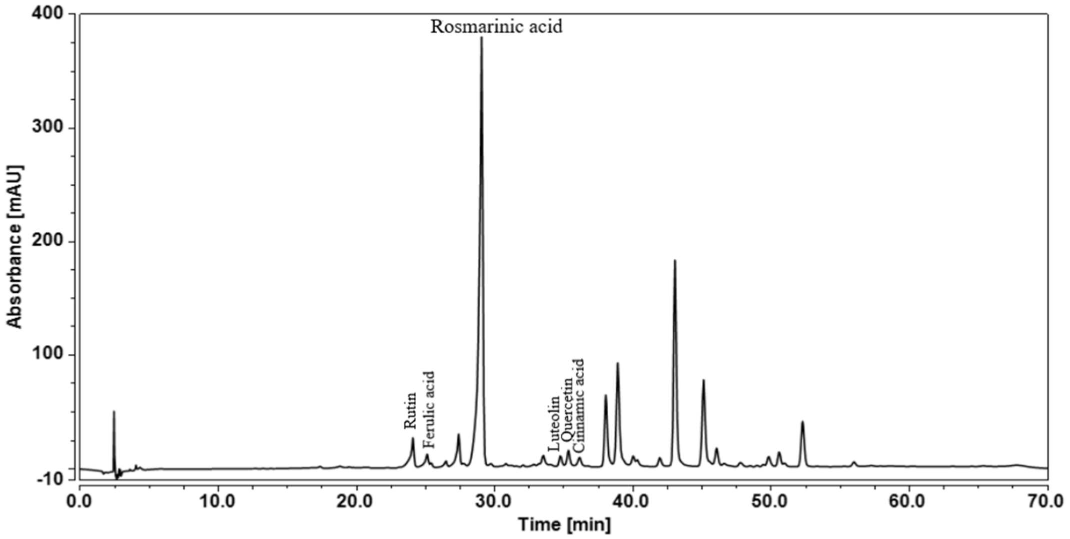

Figure S5. Chemical composition in leaves of dried Mentha analyzed by using HPLC.

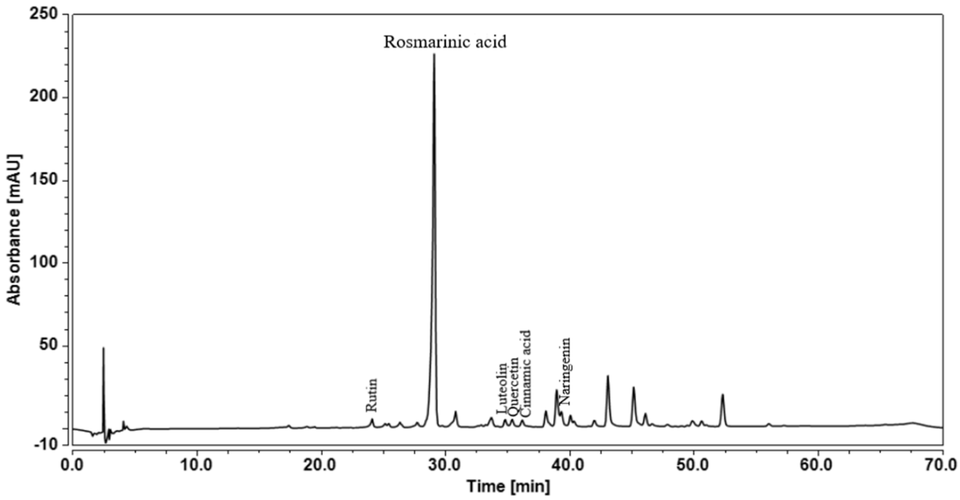

Figure S6. Chemical composition in stems of dried Mentha analyzed by using HPLC.

Effect of Volatile Oils and non-Volatile Extract on Mycelium Growth of Isolated Fungi

In total, 15 mL of PDA medium were placed in each Petri dish (9 cm diam.), and after solidification, a circular hole (2 cm, diam.) was formed. Then, 1 mL of each different concentration of the tested extract was added to each well. Each Petri dish was inoculated with four fungal species and incubated at 28°C for seven days. PDA medium without additives was used as a negative control, whereas plates containing 20 mg/mL Miconazole Nitrate were used as a positive control. Each treatment was repeated three times and subjected to statistical analysis. The diameter of inhibition zones was measured for each well, and the results were statistically analyzed .

A total of 1 mL of plant extract (20 mg/mL) was added to each well to determine the inhibition zone and inhibition percentage.(%)

The diameter of inhibition zones was measured and the percentage of antifungal activity was calculated according to Equation (2):

$$\text{Inhibition (\%)} = (DC - DT)/DC \times 100 \quad (2)$$

DC = the diameter of fungal mycelium growth in the control Petri dish .

DT = the diameter of fungal mycelium growth in the treated Petri dish, containing Mentha extracts.
